# In vivo antimicrobial activity of 0.6% povidone-iodine eye drops in patients undergoing intravitreal injections: a prospective study

**DOI:** 10.1038/s41598-021-02831-w

**Published:** 2021-12-02

**Authors:** Daniele Tognetto, Marco R. Pastore, Lorenzo Belfanti, Riccardo Merli, Alex L. Vinciguerra, Marina Busetti, Giulia Barbati, Gabriella Cirigliano

**Affiliations:** 1 Eye Clinic, Department of Medical, Surgical Sciences and Health, University of Trieste, Ospedale Maggiore, Piazza Ospitale 1, 34129 Trieste, Italy; 2 Microbiology Unit, University Hospital of Trieste, Trieste, Italy; 3 Biostatistics Unit, Department of Medical Sciences, University of Trieste, Trieste, Italy

**Keywords:** Clinical microbiology, Antimicrobial therapy

## Abstract

To investigate the antimicrobial activity of a preservative-free 0.6% povidone-iodine eye drop as an antiseptic procedure in decreasing the conjunctival bacterial load in eyes scheduled for intravitreal treatment and to compare its efficacy to the untreated fellow eye used as the control group. Prospective cohort analysis in which 208 patients received preservative-free 0.6% povidone-iodine eye drops three times a day for three days before intravitreal injection. Before and after the prophylactic treatment, a conjunctival swab was collected from both the study eye and the untreated contralateral eye, used as control. The swab was inoculated on different culture media and the colony-forming units were counted. Bacteria and fungi were identified by matrix-assisted laser desorption ionization time-of-flight mass spectrometry. Treatment with 0.6% povidone-iodine eye drops significantly reduced the conjunctival bacterial load from baseline (p < 0.001 for blood agar and p < 0.001 for chocolate agar) with an eradication rate of 80%. The most commonly isolated pathogen at each time-point and in both groups was coagulase-negative Staphylococci, isolated in 84% of the positive cultures. The study provides evidence about the effectiveness of 0.6% povidone-iodine eye drops treatment in reducing the conjunctival bacterial load in eyes scheduled for intravitreal treatment.

## Introduction

Intravitreal injections (IVI) of antivascular endothelial growth factor (VEGF) agents have become the most performed intraocular procedures in ophthalmology, with numbers that increase dramatically every year^1^. The use of anti-VEGF injections has proven to be the mainstay of treatment for several retinal diseases, including wet age-related macular degeneration (ARMD), macular edema secondary to diabetes and retinal vein occlusion, myopic choroidal neovascularization (CNV), and proliferative diabetic retinopathy^1^.

Because of the natural history of these ocular conditions and the short half-life of the drugs delivered into the vitreous, most patients require a series of anti-VEGF IVI to achieve the therapeutic effects.

The retreatment with several IVI per year exposes patients to a greater risk of infectious complications, specifically endophthalmitis^2^.

Although rare, endophthalmitis is one of the most feared ocular complications of IVI because of its rapid and aggressive evolution, often leading to poor functional prognosis if not promptly and adequately treated^3,4^. In most cases, the causative agent, most likely originates from the conjunctival bacteria flora, is directly inoculated during the perioperative phase.

Despite the absence of evidence-based data, topical antibiotics prophylaxis is an “accepted practice” related to mandatory use reported in the pivotal clinical trial protocols for IVI and it has long been the standard of care after intravitreal treatment^5^.

Recently, the real effectiveness of topical antibiotics used in preventing endophthalmitis after IVI has been questioned. Although topical antibiotics administration has been reported to reduce the conjunctival flora, antibiotic prophylaxis does not decrease post-injection endophthalmitis incidence^6^. Conversely, it may be associated with a greater risk of post-operative infections: the recurrent and short-term administration of antibiotics eye drop may promote antibiotic-resistant bacterial strains with increasing post-injection endophthalmitis rates^7^.

The use of povidone-iodine (PI), introduced in ophthalmology since the 1990s, is the most reliable form of ocular surface disinfection in the pre-operative site and is the only antiseptic procedure that allows decreasing the risk of postoperative endophthalmitis in the anterior segment surgery^8^. PI does not induce resistance and is effective against a wide spectrum of microbes^8^. According to the higher concentration of bactericidal free iodine in 5% than in 10% solution^9^, the application of 5% PI in the conjunctival sac for at least 3 min before surgery is a mandatory measure to reduce the number of bacteria in the area of the surgical wound^10,11^. Nevertheless, the PI antisepsis does not allow a complete eradication of conjunctival bacterial load with a residual incidence of endophthalmitis rate after IVI from 0.02 to 0.3% for a single injection, and a cumulative rate up to 1% after a treatment series^12^.

In the European Society of Cataract and refractive Surgeons guidelines for prevention and treatment of endophthalmitis following cataract surgery, the preservative-free 0.6% PI eye drops was lately proposed as a prophylactic treatment in the days pre-surgery to further reduce the risk of postoperative intraocular infections^10^.

A recent analysis in patients undergoing anti-VEGF IVI evaluated the efficacy of the preservative-free 0.6% PI eye drops but enrolling the study eye and the control eye from different patients^13^. However, a variation in conjunctival bacterial flora not only from patient to patient but also over time and from region to region has been demonstrated^14,15^. To the best of our knowledge, no studies report the direct comparison between treated and control eyes in the same patients, using the fellow eye as the control group.

This study aimed to investigate the antimicrobial activity of a preservative-free 0.6% PI eye drop as an antiseptic procedure in decreasing the conjunctival bacterial load in eyes scheduled for anti-VEGF intravitreal treatment and compare its efficacy to the untreated contralateral eye.

## Results

**Figure 1:**
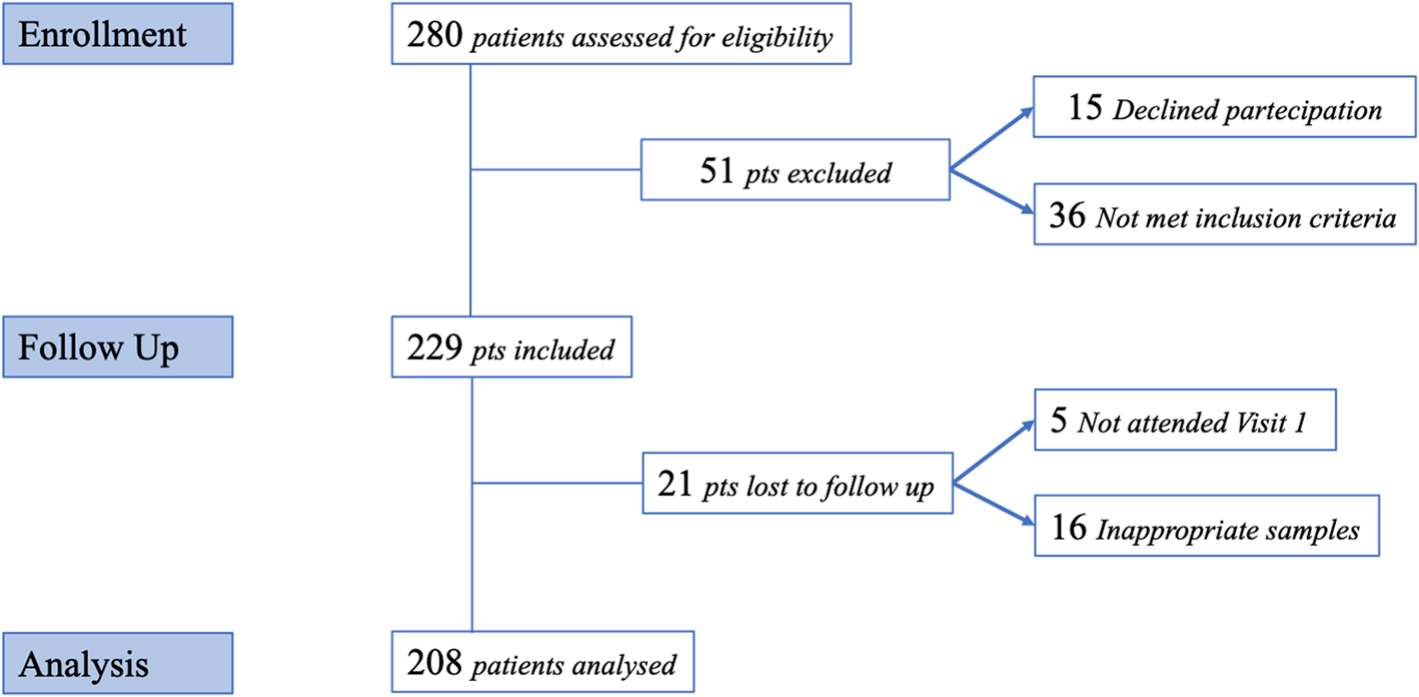
Study flow-chart.

Among the 280 patients assessed for eligibility, 15 declined to be enrolled in the study, and 36 did not met the inclusion criteria. 21 out of 229 patients admitted were lost to follow-up for inappropriate samples (16 patients) or not completed the visit V1 the day of the IVI (Fig. 1).

Among 208 patients included in the final analysis, 96 (46%) were males and 112 (54%) females. The mean age was 73.4 ± 7.6 years, ranging from 62 to 85 years. The baseline and demographic characteristics of the study cohort are summarized in Table 1.

**Table 1:** Baseline and demographic characteristics of the study cohort.

Characteristics	
Study cohort	208
Gender (man:woman)	96:112
Mean age (± SD)	73.4 (7.6)
**Diagnosis**	
wAMD	48.6%
DME	30.8%
RVO	12%
mCNV	4.8%
pDR	3.8%

*SD* standard deviation, *WAMD* wet age-related macular degeneration age-related macular degeneration, *DME* diabetic macular edema, *RVO* retinal vein occlusion, *mCNV* myopic choroidal neovascularization, *pDR* proliferative diabetic retinopathy.

In the study cohort, 48.6% (101/208) of the study eyes were scheduled to anti-VEGF intravitreal treatment for wet age-related macular degeneration, 30.8% (64/208) for diabetic macular edema, 12% (25/208) for macular edema secondary to retinal vein occlusion, 4.8% (10/208) and 3.8% (8/208) for myopic choroidal neovascularization and proliferative diabetic retinopathy, respectively.

In all 208 blood agar and chocolate agar plates seeded at V0 and V1 a microbial growth was seen. No growth was observed in Mac Conkey agar cultures and Sabouraud dextrose agar cultures with gentamicin at different time point. The CFU results at V0 and V1 on the blood agar and chocolate agar plates are reported in Table 2.

**Table 2:** Colony-forming units in the study and control group.

		Study eyes		Control eyes	
	CFU	V0	V1	V0	V1
Blood Agar	0 to 10	88	195	103	97
	11 to 100	105	13	90	98
	101 to 1000	15	0	15	13
	Mean	31.1	6.6	32.2	30.3
	SD	43.4	4.6	42.9	40.9
Chocolate agar	0 to 10	112	190	123	124
	11 to 100	86	18	72	65
	101 to 1000	10	0	13	19
	Mean	22.6	6.1	23.9	25.4
	SD	28.7	4.9	34.6	37.5

*CFU* colony-forming units, *V0* baseline visit, *V1* visit the day of intravitreal injection, *SD* standard deviation.

At V0 the mean bacterial growth was similar in the treated and control eyes in the blood agar (p = 0.344) and chocolate agar plates (p = 0.425). The treatment with 0.6% PI eye drops significantly reduced the conjunctival bacterial load in both blood and agar culture media compared to baseline (p < 0.001 blood agar V1 vs. V0, p < 0.001 chocolate agar V1 vs. V0; Table 2). In the control eyes, no statistically significant differences were found between the mean CFU at V0 and V1 (p = 0.286 blood agar V1 vs. V0, p = 0.563 chocolate agar V1 vs. V0).

As previous reported, at V0 in all 208 seeded blood and chocolate agar plates for treated and control eyes a positive culture was found. At V1, the number of positive cultures decreases to 41 (20%) in the study eyes, with a statistically significant decrease compared to V0 (p < 0.001; Table 3), and remains stable in the eyes used as control. The analysis in the study eyes demonstrated an eradication rate of 80%.

**Table 3:** Isolated bacteria in the study population.

	Study eyes		Control eyes	
	V0	V1	V0	V1
CoN Staphylococcus	180 (86%)	32 (80%)	174 (84%)	184 (88%)
Staphylococcus aureus	20 (10%)	6 (15%)	15 (7%)	15 (7%)
α-Hemolytic Streptococcus	14 (7%)	0	18 (9%)	25 (12%)
β-Hemolytic Streptococcus	5 (4%)	1 (2.5%)	14 (6%)	16 (8%)
Enterococci	10 (5%)	0	13 (6%)	10 (5%)
Corynebactyerium species	9 (4%)	0	7 (3%)	10 (5%)
Propionibacterium acnes	3 (1%)	1 (2.5%)	5 (2%)	5 (2.%)
Other Gram-negative rods	7 (3% )	0	6 (3%)	5 (2%)
Bacillus species	6 (3%)	1 (2.5%)	5 (2%)	8 (4%)

*V0* baseline visit, *V1* visit the day of intravitreal injection, *CoN* Coagulase-negative.

The most commonly isolated pathogen at each time-point and in both groups was coagulase-negative Staphylococci, isolated in 84% of the positive cultures. The treatment with 0.6% PI eyedrops did not significantly affect this ratio after the administration in the study eyes. Other bacteria identified in order of frequency were staphylococci aureus and other gram-positive bacteria, as reported in Table 3. The microbiological analysis at V0 also revealed 3% of gram-negative bacteria in both the study and control eyes. At V1 in the treated eyes, all gram-negative bacteria were eradicated.

None case of traumatic cataract, endophthalmitis, retinal detachment, or vitreous hemorrhage was reported in any of the 208 participants. No systemic adverse reactions related to the topical treatment were detected. 8 (3.9%) out of 208 patients who underwent 0.6% PI eye drops treatment experienced conjunctival hyperemia with light serous conjunctival discharge in 3 eyes (1.4%).

## Discussion

This prospective cohort study provides evidence of the effectiveness of 0.6% PI eye drops treatment in reducing the conjunctival bacterial load in eyes scheduled for IVI, compared to contralateral not treated eyes.

Ocular conjunctival flora comprises a wide range of bacteria that do not cause infection in normal conditions but can be a major contamination route during ocular surgery. Since conjunctival microorganism flora differs greatly in individuals based on age, sex, geographical distribution, and related pathologies^16^, we reported first the direct comparison of the ocular bacteria flora between treated and control eyes in the same patients, detecting a statistically significant efficacy (p < 0.001) of 0.6% PI eye drops in reducing the conjunctival bacterial load.

The IVI is an ever-increasing procedure in ophthalmology, with 20 million estimated IVI in 2016 worldwide^17,18^. The exponential growth of this treatment highlights the need to reduce possible risks and related complications. Bacteria from the patient’s conjunctiva and eyelids can be a potential source for endophthalmitis occurrence, one of the most feared postoperative complications^2–4^. The risk of endophthalmitis after IVI ranges from 0.029% to 0.057%^19^.

Topical antibiotics have broadly represented the standard of care of ocular surgery prophylaxis. Still, they have been demonstrated to have a strict correlation with a higher incidence of post-IVI endophthalmitis rate and greater antibiotic resistance^6,20–22^. A systematic review comparing the rates of endophthalmitis in eyes receiving IVI with and without topical antibiotic prophylaxis reported 74 cases of endophthalmitis in 147.203 injections using antibiotic prophylaxis compared with 55 cases in 211.418 injections with no prophylaxis. Menchini et al. concluded that antibiotic prophylaxis does not reduce the rate of endophthalmitis^21^. Similarly, a recent review detected an estimated incidence of endophthalmitis with topical antibiotic prophylaxis approximated three times the incidence without prophylaxis^6^.

Furthermore, several studies proved that the risk of endophthalmitis developing after intravitreal injection is not reduced using post-injection topical antibiotic prophylaxis but is rather associated with a trend toward a higher incidence of endophthalmitis^7,22^. According to these results, perioperative antibiotics cannot be considered the standard of care since there is no evidence of prophylactic effects regarding postoperative endophthalmitis.

Up to now, PI is the most reliable and widely used antiseptic agent for preoperative preparation in ocular surgery, being the 5% concentration recommended by the guidelines of the European Society of Cataract and Refractive Surgeons and American Academy of Ophthalmology^10,11^. Interestingly, since free iodine is not liberated readily when using high concentration solutions, diluting the solution facilitates its release^23^. Several in vitro studies and clinical studies evinced that the more diluted 0.6% preparation was more rapidly bactericidal than the 5% PI formulation, probably due to the ready release of iodine from molecular complex and therefore bactericidal activity is enhanced^23–25^.

In our study, 0.6% PI eye drops treatment showed a significant reduction of the CFU value with an eradication rate of 80% of the number of bacterial growth-positive swab cultures. Similarly, Reibaldi et al. detected a significant decrease in bacterial growth from conjunctival swab cultures after a 0.6% PI prophylaxis compared to baseline and placebo prophylaxis, used as a control group^13^.

Since in our report the control group was represented by the contralateral eye of the same subject, the bias related to a different conjunctival flora between two different subjects included in this study was eliminated.

Additionally, Reibaldi et al. found 13% positive cultures after the treatment than 20% showed in our study. These results are probably due to the more sensitive method used in our investigation to identify the bacteria, the MALDI-ToF–MS, with respect to the broth cultures of the Reibaldi et al. study.

Consistent with the literature^13–15^, the most commonly isolated organism in our analysis was coagulase-negative staphylococcus, which included the most representative Staphylococcus epidermidis, followed by Staphylococci aureus and other gram-positive bacteria. After prophylaxis with 0.6% PI, all gram-negative bacteria were eliminated. The PI in concentrations between 0.1% and 1.0% needs only 15 s to kill bacteria, but unfortunately, the duration of cytotoxic activity is short, and multiple applications are required^26,27^. Furthermore, high concentrations of PI are responsible for the toxic effect on the corneal epithelium and, therefore, not suitable for continuous administration^27^. However, in our analysis, the preservative-free 0.6% PI eye drops proved to be well tolerated by the study population.

The main limitation of this study includes the possibility that enrolled patients had not been compliant with the treatment program provided. However, according to the data, all patients proved to be compliant with the assigned treatment schedule during the baseline visit.

In conclusion, this study highlights the role of 0.6% PI eye drops in reducing the conjunctival bacterial load in eyes scheduled for IVI treatment while also showing a good safety profile. The introduction of prophylactic treatment with 0.6% PI eyedrops would limit the emerging phenomenon of antibiotic resistance and reduce the conjunctival bacterial commensal flora further limiting the still existing risk of endophthalmitis.

## Materials and methods

### Study design

A prospective cohort study was conducted at the University Eye Clinic of Trieste, a tertiary eye care center in the northeast of Italy. The study was performed in conformity with the Declaration of Helsinki and the standards of Good Clinical Practice. The Regional Ethical Committee (Comitato Etico Unico regionale, CEUR) approved the research protocol (ID 245_2019), and written informed consent was obtained from all participants.

All naïve patients affected by CNV in ARMD, diabetic macular edema, macular edema secondary to retinal vein occlusion, myopic CNV or proliferative diabetic retinopathy, and scheduled for the first IVI of anti-VEGF agents were considered eligible to be included in the study. Patients with iodine allergy, use of any eye drops or ointment within six months from enrolment, use of systemic antibiotics or corticosteroids in the last six months, active ocular and periocular infection or inflammation, previous ocular surgery (except for cataract surgery performed in the previous 12 months) were excluded from the study. Patients with cognitive impairment and unable to manage home-assigned treatment were also excluded.

All patients received, only in the eye to be injected (study eye), the preservative-free 0.6% PI eye drops (IODIM® Medivis, Catania, Italy) three times a day for three days before IVI. Patients were instructed to close their eyes for 5 min after each application. On the injection day, all study eyes received one IODIM® drop one hour before the intravitreal treatment. No treatment was administered in the fellow eye (control eye).

Before starting the IODIM® treatment (V0) and before the IVI (V1), all patients underwent a complete ophthalmologic examination, including the best-corrected visual acuity evaluation using Early Treatment Diabetic Retinopathy Study acuity charts, slit-lamp biomicroscopy, IOP measurement with Goldmann tonometer, and fundus examination. At V1 also any systemic or ocular adverse events were recorded.

### Conjunctival sample collection

At V0 and V1 visits, a conjunctival swab with ESwab™ system (Liquid Amies Elution Swab, Copan, Brescia, Italy) was collected from the study eye and the untreated contralateral one, used as control.

The ESwab™ included sterile nylon flocked swab combined with 1 mL of Liquid Amies transport medium. While the patient looked upward, the specimen collection was performed exposing the inferior fornix and twirling the mini tip flocked nylon applicator into the inferotemporal conjunctiva. Extreme attention was paid to no getting in contact with eyelids and lashes. If accidental contamination with these structures occurred, the patient was excluded from further analysis. At the end of the collection, the swab was immediately placed in the Liquid Amies transport medium and transported within two hours to the laboratory, where it was refrigerated at 4 °C and processed within 24 h.

### Microbiological analysis

The microbiologists involved in the study worked in a masked mode. In strict sterile condition at the lab, the swab was inoculated on different culture media: blood agar plate incubated at 35 °C for 72 h, chocolate agar plate incubated at 35 °C in CO_2_ enriched atmosphere for 72 h for fastidious bacteria, Mac Conkey agar plate at 35 °C for 72 h for gram-negative bacteria like pseudomonas, and Sabouraud dextrose agar plate with gentamicin at 25 °C for five days selective for fungi. If the culture was positive, the colony-forming units (CFUs) were counted in the different culture media.

Bacteria and fungi were identified by matrix-assisted laser desorption ionization time-of-flight mass spectrometry (MALDI-ToF–MS, VITEK® MS Plus)^28^.

### Statistical analysis

Statistical analyses were performed using SPSS software 11.0 (SPSS Inc, Chicago, IL). Descriptive statistics are used to report the demographic and ocular characteristics of the study population, expressed as means and standard deviation for quantitative variables, and summarized as frequency and percentage for qualitative variables. The comparison between the study and control group was performed using the non-parametric Wilcoxon paired test. Statistical significance was set at p < 0.05.

### Consent to participate

Informed consent was obtained from all individual participants included in the study.

## Data Availability

All data that supports the findings of this study are available from the corresponding author, M. R. P., upon reasonable request.
